# The aging self: how older men and women reflect on themselves and others

**DOI:** 10.3389/fnagi.2025.1600029

**Published:** 2025-06-05

**Authors:** Marta Paź, Anna Nowicka

**Affiliations:** Laboratory of Language Neurobiology, Nencki Institute of Experimental Biology of Polish Academy of Sciences, Warsaw, Poland

**Keywords:** ERP, sex differences, self-knowledge, personality traits, older adults

## Abstract

**Objectives:**

Self-relevant stimuli, such as one’s name or face, are prioritised by the brain, leading to faster responses and enhanced attentional processes. The psychophysiological underpinnings of those mechanisms are related to early and late event-related potential (ERP) components, particularly to reduced N2 and enhanced late positive potential (LPP). However, most up-to-date research on the self has focused on groups of young adults while older groups were rarely recruited for such experiments — the present study aimed to fill this gap. Thus, age-related changes in the self-referential processing were investigated and plausible gender differences were explored.

**Methods:**

EEG data were collected from 56 participants over 60 years old. Before the experiment, participants completed psychological assessments and selected a close person (e.g., a friend) and a preferred Polish celebrity (e.g., Lech Wałęsa). The experimental task consisted of three blocks (Self, Close, Famous), where participants were viewing 150 adjectives and judged whether each adjective applied to the description of the assigned person.

**Results:**

ERP results indicated higher LPP amplitudes and reduced negative N2 components in the self condition in comparison both to the close as well as famous person conditions. Notably, these effects seem to be more pronounced in older women.

**Discussion:**

Enhanced LPP indicates deeper self-reflection linked to the highest attention allocation to self-related personality traits. On the other hand, reduced N2 suggests lower involvement of executive control in visual encoding and response execution. In conclusion, the findings of our study revealed a robust self-prioritization effect in older adults, driven primarily by women.

## Introduction

The self encompasses personal identity, autobiographical memories of past events, and expectations or beliefs about the future (Frith and Frith, 2006). In exploring the concept of self, researchers typically concentrate on either its physical or psychological dimensions (Gillihan and Farah, 2005). The physical self is frequently examined through research on self-face and self-body recognition (e.g., Żochowska et al., 2023; Tacikowski and Nowicka, 2010; Lenggenhager et al., 2007), whereas the psychological self is predominantly studied in relation to self-knowledge (e.g., Nowicka et al., 2018; Tanguay et al., 2018).

The concept of self-knowledge refers to an individual’s understanding of their thoughts, feelings, motivations, behaviors, and personality traits. It includes an awareness of one’s strengths, weaknesses, values, beliefs, and desires. Gaining self-knowledge often involves introspection, reflection, and receiving feedback from others (Sedikides and Spencer, 2011). It is regarded as a dynamic and evolving process, as individuals grow, change, and adapt over time (Brown, 2014). Therefore, the capacity to evaluate one’s own personality—i.e., self-reflection—constitutes one of the essential components of human consciousness.

Numerous studies have investigated the process of self-reflection elicited by the evaluation of personality traits using various neuroimaging methods. Studies using the fMRI method provide strong evidence that the default mode network (DMN), particularly the medial prefrontal cortex (MPFC), is highly active during the processing of self-related personality traits (Gutchess et al., 2007, 2015). Event-related potential (ERP) studies have shown that reflecting on one’s characteristics is associated with the late positive potential – LPP (Nowicka et al., 2018). The LPP is a positive, sustained ERP component that begins around 400–500 ms after stimulus onset and exhibits a broad frontal-central-parietal topography (Grecucci et al., 2019). The LPP reflects a spatially non-specific (i.e., global), temporary increase in attention that facilitates the processing of the salient stimulus that elicited it (Hajcak and Foti, 2020; Brown et al., 2012). Furthermore, the LPP is linked also to the significance of stimuli that is considered as the extent to which a stimulus activates motivational systems (Lang et al., 1997). The LPP is often analyzed in the two subsequent time windows (Keil et al., 2002; Auerbach et al., 2015; Speed et al., 2016; Deng et al., 2019; Żochowska et al., 2021). The early LPP reflects initial reactivity and attentional capture by salient stimuli (Paulmann et al., 2013; Schupp et al., 2000), whereas the late LPP is linked to more regulated, sustained attention and prolonged affective processing (Langeslag and Van Strien, 2010; Ruchkin et al., 1988).

Traits perceived as highly self-descriptive are linked to greater LPP amplitudes compared to those considered less self-descriptive (Zhang et al., 2013). Furthermore, enhanced LPP amplitudes have been observed when participants made trait judgments about themselves, as opposed to making such judgments about a celebrity or a close other (Nowicka et al., 2018).

In a similar task, where subjects were asked to attribute personality traits in relation to the self and others, the analysis of an earlier ERP component – midfrontal negative component N2 – revealed smaller N2 amplitudes elicited by self-referential processing compared to non-self-referential processing (Yang et al., 2007; Liu et al., 2020). As N2 is often is considered an index of the need to exert cognitive and executive control (Folstein and Van Petten, 2008), reduced N2 may suggest lower engagement of such processes. It is worth noting that all of these studies were conducted with young adult participants.

For older populations, most studies focus on cognitive abilities that tend to decline with age, such as episodic memory, attention, executive functioning, and processing speed (e.g., Harada et al., 2013). It is important to emphasise that self-concept formation neither begins nor ends with adolescence; it is a lifelong process (Brown, 2014). Therefore, it can be assumed that some age-related changes in self-knowledge should be observed. However, fMRI studies examining self-reflection in the aging population found that the MPFC was similarly engaged by both young and older adults during self-referential judgments (Gutchess et al., 2007; Feyers et al., 2010; Gutchess et al., 2015). Nevertheless, some age-related effects were also observed. The MPFC interacted differently with other brain regions depending on age (Feyers et al., 2010). Additionally, age-related modulation was found in the dorsal prefrontal cortex. Older adults (but not young adults) showed increased activity in this area for positive relative to negative personality traits (Gutchess et al., 2007).

Taking into account that, on average, men and women differ in their self-perceptions, values, and personalities (Schwartz and Rubel, 2005), it is reasonable to expect that some sex-related differences may emerge in the self-reflection process. In line with this view, an ERP study on self- and other-related personality trait assessments revealed gender differences in LPP amplitudes among younger participants (Kotlewska and Nowicka, 2016). Specifically, in women, the LPP was enhanced in the self-condition compared to the close-other and famous person conditions, whereas these differences were absent in men.

Therefore, the aims of our study were twofold: (i) to investigate early (N2) and late (LPP) ERPs correlates associated with the processing of personality traits in the older population, and (ii) to examine whether any gender-related effects could be observed in older participants.

## Materials and methods

### Participants

Fifty-six healthy participants aged 60 to 79 years (32 females) were recruited for the study, with a mean age of 67.8 ± 4.0 years. Based on the Edinburgh Handedness Inventory (Oldfield, 1971), fifty-four participants were right-handed, while two exhibited left-handed tendencies. Cognitive functioning was assessed using the Polish version of the Montreal Cognitive Assessment (MOCA; Gierus et al., 2015, Nasreddine et al., 2005), with an average score of (M ± SD) 25.80 ± 2.37. All participants reported normal or corrected-to-normal vision and no history of psychiatric or neurological disorders, including dementia. The required sample size was estimated using the G*Power software. Estimation was conducted for a repeated measures ANOVA (estimated effect size *f* = 0.25, *α* = 0.05, *β* = 0.95, and non-Sphericity correction *ε* = 1.0), resulting in a sample size estimate of 44 participants. However, considering the potential risk of data loss or exclusion, the group size was increased to 56.

The participants were asked to fill out 6 psychological questionnaires translated into Polish: The Inclusion of Other in the Self Scale (IOS) (Aron et al., 1992), the Revised UCLA Loneliness Scale (R-UCLA) (Kwiatkowska et al., 2017; McWhirter, 1990), The Satisfaction With Life Scale (SWLS) (Diener et al., 1985), Beck’s Depression Inventory (BECK) (Zawadzki et al., 2009; Beck et al., 1996), and Rosenberg Self-esteem Scale (SES) (Łaguna et al., 2007; Robins et al., 2001). All the means across the groups, standard deviations (SD), standard errors (SE), and coefficients of variation are presented in Table 1.

**Table 1:** The descriptive statistics of all the psychological tests (IOS, BECK, UCLA, SES, MOCA, SWLS) in both experimental groups (M-men and F-female).

Test	Group	N	Mean	Std. error of mean	Std. Deviation	Coefficient of variation
IOS	F	24	5.250	0.352	1.726	0.329
IOS	M	21	5.762	0.337	1.546	0.268
BECK	F	24	10.208	1.242	6.086	0.596
BECK	M	21	5.238	1.157	5.300	1.012
UCLA	F	24	32.958	2.263	11.087	0.336
UCLA	M	21	33.632	1.376	6.304	0.187
SES	F	24	9.875	0.606	2.968	0.301
SES	M	21	8.571	0.668	3.059	0.357
MOCA	F	24	25.792	0.493	2.413	0.094
MOCA	M	21	26.238	0.478	2.189	0.083
SWSL	F	24	20.792	1.383	6.776	0.326
SWSL	M	21	27.190	0.572	2.620	0.096

Nine participants were excluded from the final analysis: seven due to excessive EEG artefacts resulting in an insufficient number of epochs after preprocessing, one because this person did not understand the assignment, and one for falsifying their age. Therefore, the final sample consisted of 47 participants (22 men and 25 women).

The Ethics Committee of Jagiellonian University, Krakow, Poland approved the study. Written informed consent was obtained from all participants, who received monetary compensation of 150 PLN for their participation.

### Stimuli

The set of stimuli consisted of three lists of adjectives linked to personal traits. The adjectives were adapted from Anderson’s List of Personality-Trait Words in English (Anderson, 1968) and translated into Polish.

The stimuli lists used in the experimental procedure were assigned to three conditions: Self, Close, and Famous. These assignments were randomised at the group level: each stimulus list was randomly chosen for the condition. Each of the lists included 50 unique adjectives (20 positive, 10 negative and 20 neutral) resulting in 150 adjectives. As in Polish the endings of traits depend on the sex (e.g., for men—dobry, for women—dobra), the lists of adjectives were suited for the sex of the participants, and the people they chose as their close and well-known people. Moreover, the order of traits on the lists was pseudorandomised hence more than three words with the same valence did not occur and also words were balanced with a number of letters.

### Procedure

Before starting the experiment all the participants signed the documents stating their participation was voluntary and that they could terminate at any time. Then they filled out psychological questionnaires and started the experimental procedure which was written in the Presentation software (Neurobehavioral Systems, Albany, CA, United States) and presented on a FlexScan EV-2450 (Hakusan, Ishikawa, Japan) screen through an Intel Core i3 computer. Words were displayed with white letters against a black background, with a stimuli size ranging from 3° × 1° to 11° × 1°. Participants were seated in an acoustically shielded dark room at a distance of 50 cm from the screen.

**Figure 1:**
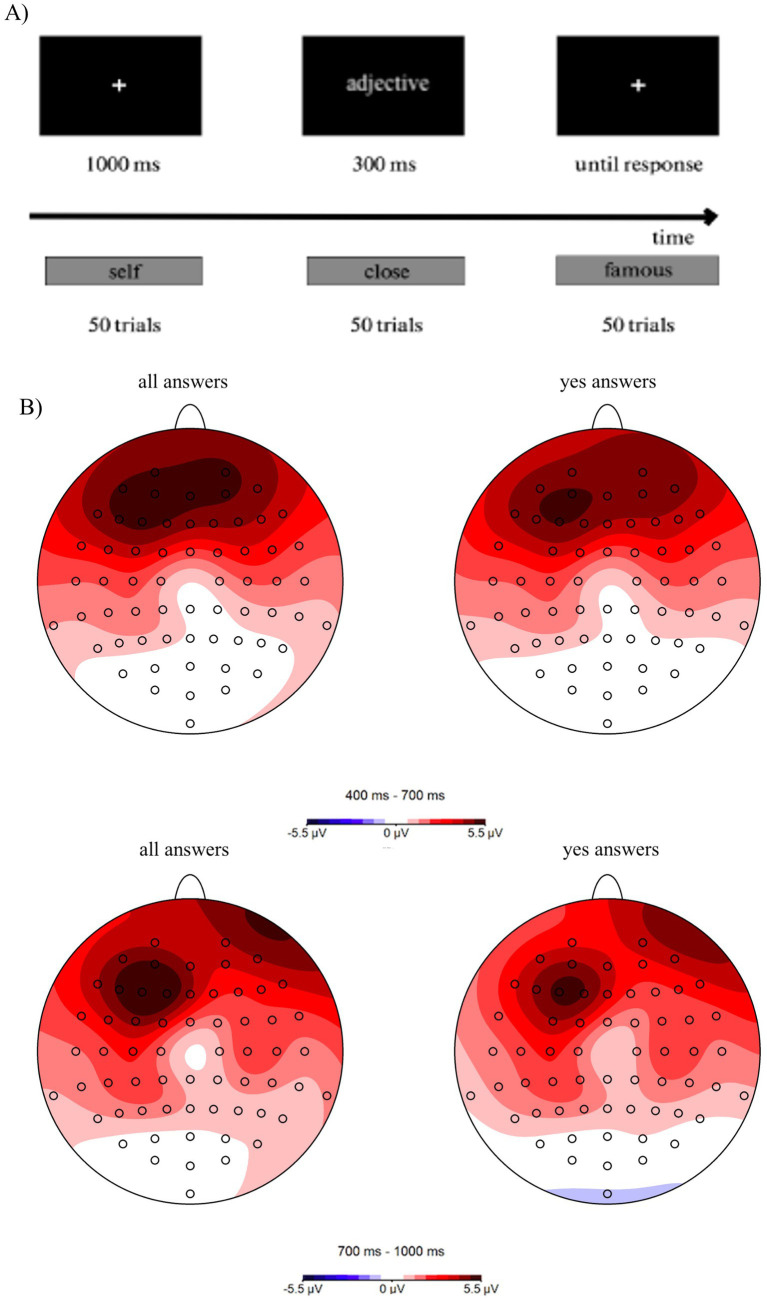
Schematic presentation of the experimental procedure **(A)** Black rectangles represents scheme of each block, meanwhile, grey rectangles represents three different blocks of experiment. Topography of early (400–700 ms) LPP of all responses and “yes” responses, collapsed across all participants and all experimental conditions (**B**, upper panel). Topography of late (700–1,000 ms) LPP of all responses and “yes” responses, collapsed across all participants and all experimental conditions (**B**, lower panel).

The experimental procedure started with instruction slides providing all the information about the study. Participants were introduced to the specifics of the answering process in the preliminary trials. The procedure consisted of three blocks, where each one was attributed to one of three conditions- Self, Close or Famous (Figure 1A). The order of blocks was randomized on the group level. Participants were asked to decide whether the adjective matched or did not match the person indicated at the beginning of the block. Subjects responded by pressing one of two buttons on a Cedrus response pad (RB-830, San Pedro, United States), using the index and middle fingers of the right hand to press keys. Key assignment to “yes” and “no” responses was counterbalanced across subjects.

A single experimental trial consisted of the following sequence of events: a black screen was presented for 1,000 ms, an adjective was presented for 300 ms, followed by a black screen displayed for 20,000 ms. Participants were asked to respond either when the word was presented or when the black screen was displayed.

### EEG recording

EEG was recorded from 62 electrically shielded scalp electrodes and two additional electrodes placed on the left and right earlobes. 128-channel amplifier (Quick Amp, Brain Products, Enschede, Netherlands) and BrainVisionRecorder^®^ software (Brain Products, Gilching, Germany) were used for collecting the EEG signal. Ag-AgCl electrodes were mounted on an elastic cap (ActiCAP, Munich, Germany) and located according to the extended 10–20 system. The impedance of the signal was kept below 5 kΩ with a sampling rate of 500 Hz.

### Analysis of behavioral data

The reaction times (RT) of trials with “yes” and “no” responses were analyzed. All analyses were conducted using custom-made Python scripts and SPSS (Version 29.0.2.0, IBM Corporation). The only responses taken under consideration were the first answers that occurred after the stimuli presentation. Mean number of “yes” and “no” responses and mean RTs from these trials were analyzed using a mixed repeated-measures ANOVA with condition (Self, Close, Famous) and type of response (“yes”, “no”) as within-subject factors and sex as a between-subject factor.

### Analysis of EEG data

The ERP analysis was conducted using BrainVisionAnalyzer^®^ software (Brain Products, Gilching, Germany). The EEG data were re-referenced to the average signal from the left and right earlobes to facilitate comparison with previous studies on self-referential processing (Nowicka et al., 2018; Kotlewska and Nowicka, 2016; Tacikowski and Nowicka, 2010). Butterworth zero-phase filters were applied: high pass (1 Hz, 12 dB/oct), low pass (30 Hz, 24 dB/oct), and a 50 Hz notch filter. Ocular artefacts were corrected using Independent Component Analysis (ICA), with eye blinks and movements visually inspected and rejected. Data were segmented into 1,5-s epochs, time-locked to stimulus onset [−200, 1,300 ms], and an automatic artefact rejection process excluded amplitudes exceeding ±50 mV. The maximal voltage step per sample was set to 50 μV, with a maximum difference of 100 μV within a 200 ms interval, and the minimal activity allowed in a 100 ms window was 0.5 μV. Two kinds of trials were taken into further analysis. One with “yes” responses, as these indicated agreement with the descriptor for the target (Self, Close, Famous) and the other—with all the given responses (both “no” and “yes”). Averaging the signal and baseline correction were performed separately for each condition. The number of valid segments used to compute ERPs did not significantly differ across conditions.

The N2 is a vertically tilted negative component typically analyzed at mid-frontal electrode locations (Fz and FCz: Żochowska and Nowicka, 2024; Fz: Guan et al., 2014). In the present study, mean amplitudes of N2 were analyzed at Fz in the 220–370 ms time window. This time window was selected based on the grand average ERP collapsed across all experimental conditions and both sexes.

**Figure 2:**
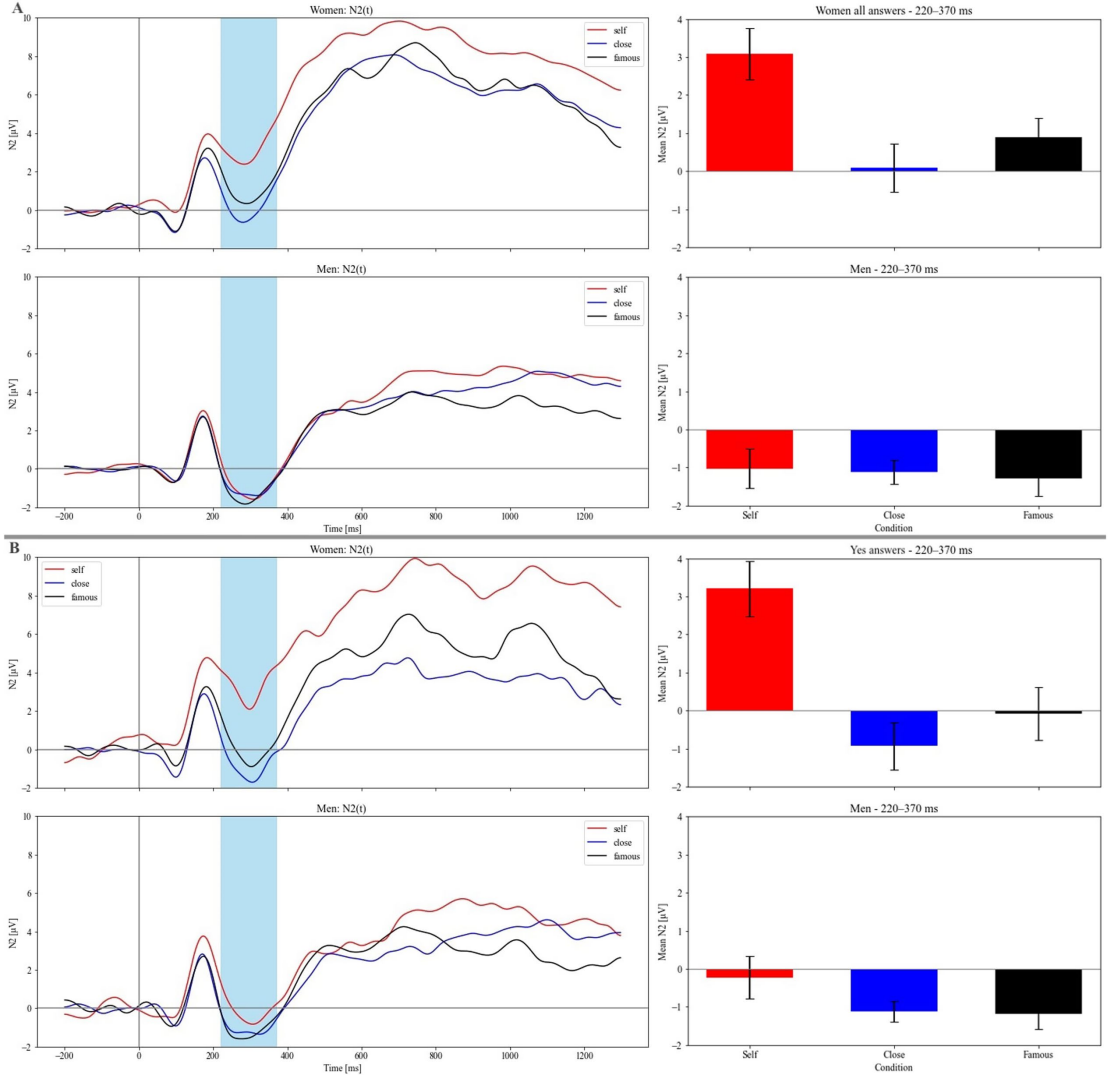
Grand-averaged N2 at the frontal region (Fz) for all responses in men (lower panel **A**) and women (upper panel **A**). Grand-averaged N2 at the frontal region (Fz) for “yes” responses in men (lower panel **B**) and women (upper panel **B**). Light-blue rectangles mark the analyzed time window. Bar charts show mean N2 amplitudes in the 220–370 time window.

In the case of the LPP, we adopted the collapsed localizer approach (Luck and Gaspelin, 2017) to select electrodes for LPP analysis in an unbiased manner. Thus, topographical activity distribution maps within the LPP time windows (400–700 ms and 700–1,000 ms) were aggregated across all experimental conditions: Self, Close, and Famous and across men and women (Figure 2). For further analyses, the electrodes AF3, AF7, F1, and F3 within the maximal activity in the frontal region were selected and pooled. The mean LPP amplitudes were analyzed in the early (400–700 ms) and late (700–1,000 ms), as this ERP component exhibits sustained activity (Hajcak and Foti, 2020) (Figure 1B). This approach has been adopted in several earlier studies (e.g., Brown et al., 2012).

Statistical analyses were focused on N2 and LPP amplitudes for all trials (“yes” and “no” responses) as well as for “yes” responses only. ERPs for all responses across the entire group of participants reflect changes in brain activity associated with the process of evaluating themselves and others. On the other hand, the LPP for the “yes” responses across all participants indicates a conscious decision regarding the self- and other-descriptiveness of the presented adjectives.

The N2 and LPP components of the ERP were analyzed using repeated-measures ANOVAs with condition (Self, Close, Famous) as a within-subject factor and sex as a between-subject factor. The normality of N2 and LPP amplitude distributions was checked with the Shapiro–Wilk test, the homogeneity of variances—with Levene’s test, and sphericity of N2 and LPP amplitude – with the Mauchly test. If the test of Sphericity indicated a violation the Greenhouse–Geisser correction was applied.

When the condition × sex interaction was significant, further analyses of condition effects were conducted separately for men and women, with Bonferroni correction applied. When the main effect of condition was significant, pairwise comparisons between conditions were performed. Statistical analyses were conducted using JASP software (JASP Team, 2024).

## Results

### Behavioral results

The mean number of “yes” responses in each experimental condition was as follows (Mean ± SD): Self Women – 26.069 ± 4.069, Self Men – 25.800 ± 5.207, Close Women – 25.241 ± 3.851, Close Men – 25.050 ± 4.186, Famous Women – 25.621 ± 3.570, and Famous Men – 25.450 ± 3.236. Meanwhile, the mean number of “no” responses in each experimental condition are as outlined (Mean ± SD): Self Women – 23.897 ± 4.100, Self Men – 24.200 ± 5.207, Close Women – 24.759 ± 3.851, Close Men – 24.950 ± 4.186, Famous Women – 24.103 ± 3.609, and Famous Men – 24.550 ± 3.236. A mixed model repeated measures model ANOVA showed a non-significant main effect of condition (F_(2, 46)_ = 0.695, *p* > 0.99, *η_p_^2^* = 0.029) and type of response (F_(1, 47)_ = 1.921, *p* > 0.99, *η_p_^2^* = 0.039). The between-subject factor (sex) was also nonsignificant (F_(1, 47)_ = 1.067, *p* > 0.99, *η_p_^2^* = 0.022). All interactions were also non-significant (*p > 0*.99).

The average RTs of “yes” responses in each experimental condition were as follows (Mean ± SD): Self Women – 1233.957 ± 441.580, Self Men – 1422.165 ± 583.601, Close Women – 1171.362 ± 295.399, Close Men – 1274.569 ± 304.430, Famous Women – 1178.519 ± 415.092, and Famous Men – 1195.818 ± 253.366. In turn, the average RTs of trials with “no” responses for each experimental condition were as detailed below (Mean ± SD): Self Women – 1234.166 ± 356.573, Self Men – 1442.933 ± 445.169, Close Women – 1213.125 ± 352.732, Close Men – 1327.251 ± 306.541, Famous Women – 1219.101 ± 383.954, and Famous Men – 1204.941 ± 273.957. A mixed model repeated measures ANOVA conducted on mean RTs yielded nonsignificant main effects of condition (F_(1, 43)_ = 1.375, *p* > 0.99, *η_p_^2^* = 0.031), type of response (F_(2, 42)_ = 2.538, *p* = 0.91, *η_p_^2^* = 0.108), and sex (F_(1, 43)_ = 1.370, *p* > 0.99, *η_p_^2^* = 0.031). All interactions were also nonsignificant (*p* > 0.99).

### ERR results

**Figure 3:**
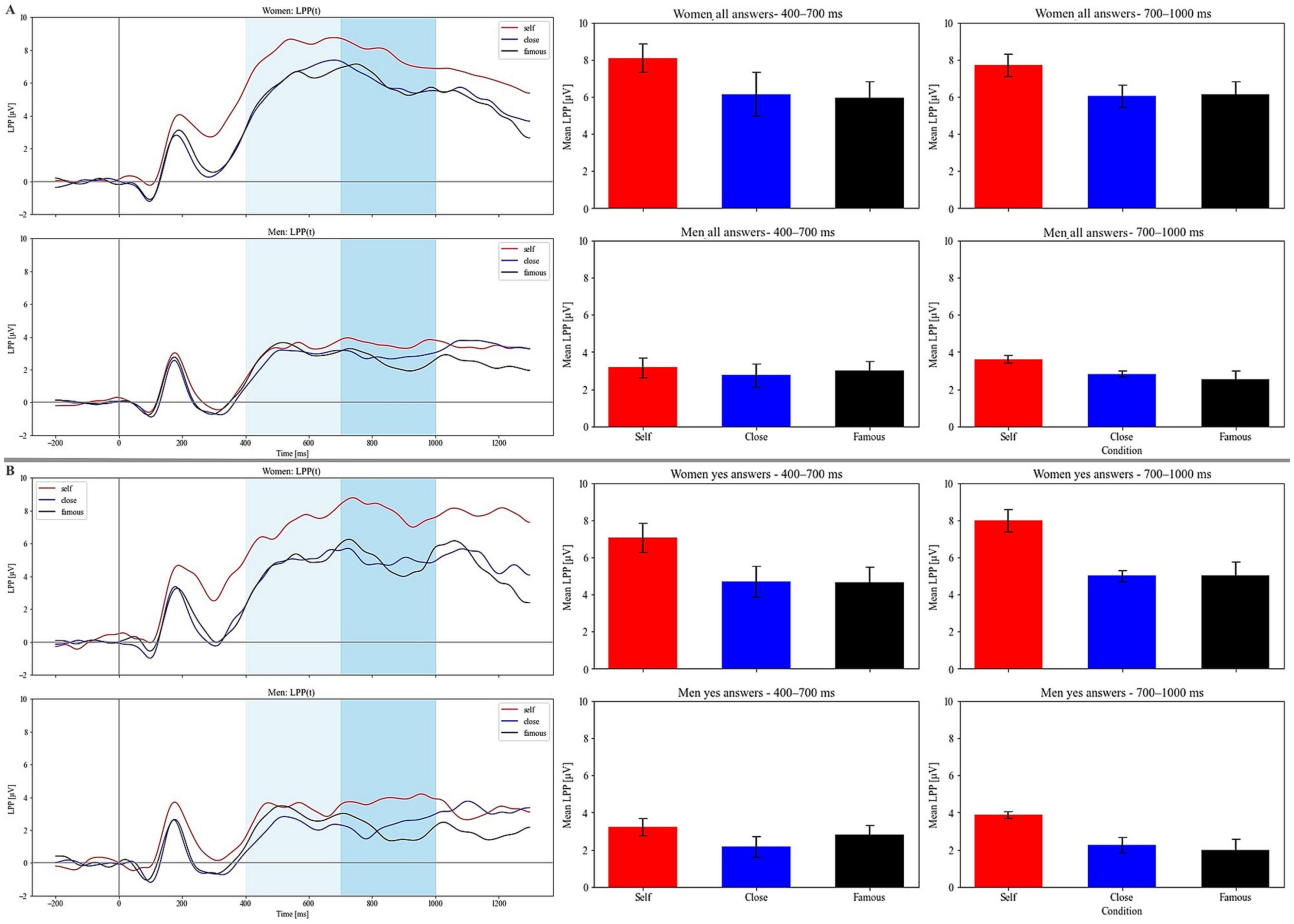
Grand-averaged LPP at the frontal region (pooled AF3, AF7, F1, F3) for all responses in men (lower panel **A**) and women (upper panel **A**). Grand-averaged LPP at the frontal region (pooled AF3, AF7, F1, F3) for “yes” responses in men (lower panel **B**) and women (upper panel **B**). Light-blue rectangles mark the analyzed time window. Bar charts show mean LPP amplitudes in the 400–700 ms and 700–1,000 ms time windows.

Figure 2 presents a grand-averaged N2 (at Fz) for all responses for men (lower panel A) and women (upper panel A) and for “yes” responses for men (lower panel B) and women (upper panel B). Figure 3 presents a grand-average LPP (at pooled AF3, AF7, F1, and F3) for all responses for men (lower panel A) and women (upper panel A) and “yes” responses for men (lower panel B) and women (upper panel B).

### N2—all responses

Repeated measures ANOVA revealed significant effects of condition (F(2, 90) = 6.369, *p* = 0.003, *η_p_^2^* = 0.026) and sex (F(1, 45) = 6.138, *p* = 0.017, *η_p_^2^* = 0.092). Moreover, the interaction between these factors was also significant (F(2, 90) = 5.617, *p* = 0.005, *η_p_^2^* = 0.023). The significance of the sex factor indicated that N2 amplitudes were more negative in men compared to women (Figure 2). Pairwise comparisons for the main factor of condition revealed significant differences between Self and Close (*p* = 0.005) and Self and Famous (*p* = 0.008), indicating reduced N2 amplitudes for Self. However Close was not significantly different from Famous (*p* = 0.432). *Post hoc* tests for the condition × sex interaction revealed significant differences between conditions only for women: Self vs. Close (*p* = 0.001) and Self vs. Famous (*p* = 0.011). These differences in the men’s group were nonsignificant (*p* > 0.999).

### N2 – “yes” responses

Repeated measures ANOVA revealed significant main effects of condition (F(2, 90) = 6.147, *p* = 0.003, *η_p_^2^* = 0.044) and sex (F(1, 45) = 6.138, *p* = 0.017, *η_p_^2^* = 0.092). However, the interaction between these factors was not significant (*p* = 0.133). Pairwise comparisons for the condition factor showed significant differences between Self and Close (*p* = 0.006) as well as between Self and Famous (*p* = 0.005), while no significant difference was found between Close and Famous (*p* = 0.306). The significance of the sex factor indicated that N2 amplitudes were more negative in men compared to women (Figure 2).

### Early LPP (400–700 ms)—all responses

The analysis of early LPP amplitudes revealed significant main effects of condition (F(2, 90) = 3.367, *p* = 0.039, *η_p_^2^* = 0.015) and sex (F(1, 45) = 13.190, *p* < 0.001, *η_p_^2^* = 0.177). The interaction between condition and sex was not significant (*p* = 0.128). The significant effect of the sex factor indicated that LPP amplitudes were generally higher in women than in men (Figure 3). However, the significance of the condition factor indicated that LPP amplitudes in both sexes were higher when evaluating adjectives in reference to the Self compared to relating them to a Close or Famous person (*p* = 0.032 and *p* = 0.040, respectively). However, the pairwise comparison of Close vs. Famous conditions was nonsignificant (*p* = 0.881).

### Late LPP (700–1,000 ms)—all responses

A similar pattern of results was observed in the case of late window LPP. Repeated measures ANOVA showed the significance of condition (F(2, 90) = 3.605, *p* = 0.031, *η_p_^2^* = 0.017) and sex (F(1, 45) = 10.173, *p* = 0.003, *η_p_^2^* = 0.143). The interaction was not significant in this case either (*p* = 0.734). LPP amplitudes were in general higher in women than in men (Figure 3). Amplitudes of LPP were also enhanced when participants evaluated themselves compared to a close and a famous person (*p* = 0.029 and *p* = 0.028, respectively). The pairwise comparison between the Close and Famous conditions was again nonsignificant (*p* = 0.728).

### Early LPP (400–1,000 ms) – “yes” responses

The results for the late LPP for “yes” responses were comparable to its equivalent in all responses. Specifically, a repeated measures ANOVA demonstrated significant main effects for condition (F(2, 90) = 3.807, *p* = 0.026, *η_p_^2^* = 0.025) and sex (F(1, 45) = 12.973, *p* < 0.001, *η_p_^2^* = 0.150). In general, amplitudes of early LPP in women were higher than in men. However, the interaction between condition and sex was nonsignificant (*p* = 0.226). LPP amplitudes were higher for self-descriptive words compared to words judged as suitable to describe a Close or Famous person (Figure 3). Pairwise comparisons identify significant differences between these conditions: Self vs. Close (*p* = 0.005) and Self vs. Famous (*p* = 0.024). However, Close did not differ from Famous (*p* = 0.999).

### Late LPP (700–1,000 ms) – “yes” responses

The results for the late-window LPP followed a similar pattern. A repeated measures ANOVA revealed significant main effects of condition (F(2, 90) = 3.770, *p* = 0.027, *η_p_^2^* = 0.028). However, the sex factor was nonsignificant (F(1, 45) = 0.036, *p* = 0.849, *η_p_^2^* < 0.001). Moreover, as before, the interaction between condition and sex was also nonsignificant (*p* = 0.823). LPP amplitudes were higher for words judged as self-related compared to words judged as characterizing a Close or Famous person (Figure 3). Pairwise comparisons showed significant differences between the following conditions: Self vs. Close (*p* = 0.003) and Self vs. Famous (*p* = 0.017). As in the earlier analyses, the Close vs. Famous comparison was nonsignificant (*p* = 0.879).

## Discussion

The majority of research involving older adults has focused on age-related changes in brain function and neurological conditions such as Alzheimer’s disease, mild cognitive impairment, and other forms of cognitive decline (e.g., Li et al., 2009; Damoiseaux, 2012; Kanda et al., 2017; Jann et al., 2023; Zhu et al., 2023a, 2023b). While these studies have significantly advanced our understanding of pathological aging, relatively fewer investigations have explored self-referential processing in healthy older individuals (e.g., Gutchess et al., 2007; Dulas et al., 2011; Tanguay et al., 2022). This represents a notable gap, given that self-referential processing plays a key role in maintaining a coherent sense of identity, emotional regulation, and decision-making across the lifespan. The present ERP study aimed to investigate sex differences in the self-reflection process among older participants. To elicit this process, participants evaluated personality traits in relation to themselves, a close other, and a famous person. The latter two experimental conditions served as control conditions, representing personally relevant and personally irrelevant contexts, respectively.

On the behavioral level, we found that in both sexes the time required to decide that presented trait adjectives were suitable to characterize one’s own person, as well as a close and famous person was similar. This is in line with previous studies (Nowicka et al., 2018). However, differences between experimental conditions were observed on the neural level.

Analyses of ERP data focused on the N2 and LPP components, which have been previously reported in studies on self-referential processing involving personality traits (Zhang et al., 2013; Nowicka et al., 2018; Liu et al., 2020). These ERP components were analyzed in two ways: (i) for all responses (i.e., combining “yes” and “no” responses) and (ii) specifically for “yes” responses. While the former analysis was associated with the processes of self-reflection and reflection on others (close or famous persons), the latter focused on the conscious decision that certain personality traits were suitable to characterize oneself or others. Overall, both analyses revealed analogous patterns of results.

For the self-referential condition, the N2 was reduced compared to the close and famous person conditions. This finding is in line with earlier studies investigating the processing of different types of self-referential information in young adults. In these studies, similar pattern of N2 differences between self- and control conditions were found for one’s own name (Guan et al., 2014; Kotlewska et al., 2023), face (Żochowska et al., 2023), and personality traits (Liu et al., 2020). It is worth noting that in the present study, this effect was driven mainly by women, as in this group differences between Self and Close/Famous person conditions were more pronounced and highly significant.

An increase in the mid-frontal N2 often signifies a more pronounced involvement of certain forms of executive control (Folstein and Van Petten, 2008). Therefore, a smaller stimulus-related amplitude increases of the mid-frontal N2 under self-related conditions, compared to the self-unrelated conditions, may suggest a reduced engagement of executive control in visual encoding and response execution (Folstein and Van Petten, 2008). Moreover, the less negative N2 in women than in men may indicate lower engagement of cognitive and executive control when reflecting on the self and others, as well as when deciding whether certain traits were suitable to characterize the self, a close other, or a famous person.

Moreover, the N2 component may also be sensitive to the processing of emotional information (Lewis et al., 2006; Stieben et al., 2007). However, the relationship between the N2 and emotional information is inconsistent and appears to depend on various factors, including the nature of the stimuli and the specific task demands. While some studies have reported increased N2 amplitudes in response to emotional stimuli (Carretié et al., 2004), others have found decreased amplitudes (Albert et al., 2010). Thus, interpreting a less negative (decreased) N2 associated with the processes of self-reflection, as well as the processing of words judged as self-descriptive, remains challenging in the context of emotional processing.

Results of the current study clearly also showed in older adults that amplitudes of both early and late LPP were modulated by the target of reflection. In the whole group of older participants, the larger amplitudes of early and late LPP were observed during the self-referential processing compared to the close and famous person conditions. However, the latter two conditions did not differ. This pattern of results was observed both when participant were reflecting about the self and others as well as when they decided that certain traits were suitable to describe the target of reflection. Enhanced LPP for the self condition is in line with previous studies conducted with the participation of young adults which consistently reported larger LPP when participants make judgments about themselves compared to making judgments about others (Zhang et al., 2013; Kotlewska and Nowicka, 2016; Nowicka et al., 2018).

In general, the LPP is commonly associated with the processing of emotional stimuli in comparison to neutral visual stimuli (Foti and Hajcak, 2008). Greater LPP amplitudes have been linked to heightened arousal (Cuthbert et al., 2000). Additionally, it has been suggested that the LPP reflects not only the emotional content of stimuli but also their overall significance, which is determined by the extent to which a stimulus activates motivational systems (Fields, 2023). Although significance is closely connected to emotional properties, it is also shaped by individual differences, contextual factors, and self-relevance, all of which can influence LPP amplitude (Hajcak and Foti, 2020). Considering this, the heightened LPP observed in the self-referential condition likely reflects the stronger perceived significance of self-referential stimuli. Furthermore, this finding may suggest that, for older adults, the process of self-evaluation as well as self-related information (e.g., self-descriptive words) evoked a certain level of emotional arousal.

Moreover, it is well documented that the LPP reflects a transient, spatially non-specific increase in attention, which facilitates the processing of salient stimuli (Brown et al., 2012). In this context, the LPP findings suggest that the highest attentional resources were allocated to the process of self-reflection and self-related personality traits, with the lowest attention directed toward both personally relevant and personally irrelevant individuals.

In addition, LPP amplitudes were generally higher in women than in men. This was evident in both the early and late LPP time windows during reflection on the self and others (i.e., trials with “yes” and “no” responses). Enhanced early and late LPP in women may be linked to increased initial reactivity and attentional capture by personality traits as well as increased sustained attention and prolonged processing of those stimuli (Paulmann et al., 2013; Langeslag and Van Strien, 2010; Schupp et al., 2000; Ruchkin et al., 1988).

However, when traits were judged as suitable to characterize the self and others (i.e., trials with “yes” responses), sex-related results differed for the early and late LPP. Specifically, the LPP was enhanced in women compared to men only in the early time window, indicating increased initial reactivity and attentional capture by those traits (Paulmann et al., 2013; Schupp et al., 2000). Figure 3 seems to suggest that LPP differences between conditions, particularly for the self condition, were much more pronounced in women than in men, although no significant interaction between sex and condition was found. This hypothesis was tested by additional analyses (see Supplementary Material) and, to some extent, confirmed, as significant differences between the Self and Close/Famous conditions were observed only in women and they were – in general – nonsignificant in men. In addition, differences between men and women were found for early and late LPP when sex groups were directly compared.

Our N2 and LPP findings for self-referential processing clearly showed that, in older adults, the self-condition differed to a similar extent from both the Close and Famous person conditions. Moreover, the N2 and LPP amplitudes for a Close and a Famous person were similar, and in each analysis, the differences between them were nonsignificant. The lack of differences between these two conditions may indicate that reflection on a personally relevant other was not associated with emotional processing and did not lead to increased attention allocation.

However, these two control conditions differed in young adults (e.g., Kotlewska and Nowicka, 2016). Therefore, it can be hypothesized that, among older participants, the three experimental conditions were distinguished based on the “me” vs. “not me” rule. This hypothesis aligns with the notion that older adults are more influenced by self-related stimuli than younger participants (Mather and Carstensen, 2005; Trelle et al., 2015) and is consistent with lay reports that older individuals become more self-absorbed and introspective (Sui and Humphreys, 2017).

One of the primary limitations of this study was the relatively low number of stimuli/trials per experimental condition, which constrained various aspects of the data analysis. Specifically, the ERP analysis could not include the valence of words. More studies are definitely needed to enable drawing stronger conclusions regarding the impact of age on the neural correlates associated with the process of self-reflection in the older population.

In conclusion, the findings of the present study revealed a strong self-prioritization effect in older adults, predominantly driven by women. Furthermore, among the older participants, information about a close person was not perceived as particularly significant or emotional and did not elicit increased attentional processing.

## Data Availability

The datasets presented in this study can be found in online repositories. The names of the repository/repositories and accession number(s) can be found at: OSF: https://osf.io/kecx7/?view_only=58c8049d35144954bd780defb41c11eb.
