# Supplementary material for: The aging self: how older men and women reflect on themselves and others
Source: Front Aging Neurosci. 2025 Jun 5;17:1600029. doi: 10.3389/fnagi.2025.1600029 (PMC12176846; doi:10.3389/fnagi.2025.1600029)
Supplement: Supplementary file 1 [file Table_1.docx]

Supplementary Material

Due to the lack of ERP studies investigating the impact of sex on self-referential processing in the older population, planned comparisons were conducted in addition to the main analyses to examine sex differences for each experimental condition. This analysis led to the conclusion that, despite the absence of significant interactions between conditions and sex in LPP results in the main analysis, the significant effects were primarily driven by women. Pairwise t-test comparisons were conducted for each LPP window (early: 400–700 ms and late: 700–1000 ms) to compare each condition (Self, Close, Famous) within both sex groups separately and for each condition between men and women. In case of normality deviation instead of t-test, Mann-Whitney test was conducted.

*Early LPP (400-700 ms)*

In the early LPP window for all-response trials, the results revealed significant differences among women between Self and Close (t(24) = 2.340, p = .028) and Self and Famous (t(24) = 2.183, p = .039). The difference in means between the Close and Famous conditions was nonsignificant (p = .958). All comparisons conducted among men were non-significant (p > .1).

Women differed from men in all conditions: Self – U(45) = 433.000, p < .001, Close – t(45) = 2.981, p = .005, Famous - t(45) = 2.919, p = .005.

In trials considering only 'yes' responses, significant differences were also observed in the women's group: between Self and Close (t(24) = 3.207, p = .004) and Self and Famous (t(24) = 2.585, p = .016). However, in this case, the difference between Close and Famous was again nonsignificant (p = .704). As in the previous analysis, the men's group did not reveal any significant differences (p > .1).

For trials with ‘yes’ responses, women also differed from men in all conditions: Self – U(45) = 429.000, p = .001, Close – t(45) = 2.844, p = .007, Famous - t(45) = 2.376, p = .022.

*Late LPP (700-1000 ms)*

In the late LPP window for all-response trials, the planned comparisons did not reveal significant differences among women, except for one result at the trend level (Self vs. Close: t(24) = 1.998, p = .057). All other results in women were non-significant (p > .1). Similarly, for men, the difference in means between the Self and Famous conditions was also at the trend level (t(21) = 1.783, p = .089), but none of the other comparisons reached significance (p > .1).

Women differed from mean in all conditions: Self – t(45) = 2.707, p = .010, Close – t(45) = 2.506, p = .016, Famous - t(45) = 3.203, p = .002.

In trials that included only 'yes' responses, significant differences were observed in the women's group: between Self and Close (t(24) = 2.920, p = .007). The difference between Self and Famous was at the trend level (t(24) = 1.739, p = .095). As in the early window trials, the differences in the men’s group were not significant (p > .1), except for the comparison between Self and Famous, which revealed a significant difference (t(24) = 2.141, p = .044).

For trials with ‘yes’ responses, women did not differ from men in all conditions.
